# Comparison of patellar anchor fixation graft and graft through patellar tunnel reconstruction of medial patellofemoral ligament in the treatment of recurrent patellar dislocation: A protocol for a meta-analysis of comparative studies

**DOI:** 10.1097/MD.0000000000032467

**Published:** 2023-01-20

**Authors:** Jian Tian, Jingfan Yang, Wei Dong, Xiaoyan Zhang, Weitong Liu, Jiankun Chen, Hong Yin, Xing Zhou, Changfei Yuan, Jinlei Li

**Affiliations:** a Youyang Hospital, A Branch of The First Affiliated Hospital of Chongqing Medical University, Chongqing City, China; b Kunming Municipal Hospital of Traditional Chinese Medicine, Kunming City, China; c Kunming University of Science and Technology Hospital, Kunming City, China; d The First Affiliated Hospital of Zhejiang Chinese Medical University, Hangzhou City, China.

**Keywords:** anchor fixation, medial patellofemoral ligament, protocol, recurrent patellar dislocation, through patellar tunnel

## Abstract

Recurrent patellar dislocation (RPD) occurs in people who have their own patellofemoral dysplasia and who have not been properly treated after their first patellar dislocation. For RPD where conservative treatment is ineffective, medial patellofemoral ligament (MPFL) reconstruction is the first choice for surgical treatment, but there are various and controversial ways of MPFL reconstruction and fixation. Initially, more scholars adopted the patellar lateral tunneling (PT) approach to contain and stabilize the graft, but with the newer materials and techniques, some experts adopted the lateral patellar anchor fixation (AF) of the graft, which can avoid the collateral damage caused by the patellar lateral tunneling and can obtain the same definite efficacy. Therefore, a meta-analysis must be performed to provide evidence whether there is a difference between AF and PT reconstruction of the MPFL in the treatment of RPD.

**Methods:**

We will search, with no time restriction, without any restriction of language and status, the time from the establishment of the database to October 2022, on the following databases: PubMed (MEDLINE), Cochrane Central Register of Controlled Trials (CENTRAL), Web of Science, Chinese National Knowledge Infrastructure (CNKI), Wanfang Data (WF), Chinese Scientific Journals Database (VIP), and Chinese databases SinoMed (CBM) electronic databases. The electronic database search will be supplemented by a manual search of the reference lists of included articles. We will apply the risk-of-bias tool of the Cochrane Collaboration for randomized controlled trials to assess the methodological quality. Risk-of-Bias Assessment Tool for Non-randomized Studies was used to evaluate the quality of comparative studies. Statistical analysis will be conducted using RevMan 5.4 software.

**Results:**

This systematic review and meta-analysis will evaluate the functional outcomes of the two fixation modalities, AF and PT, in reconstructing MPFL for RPD.

**Conclusion:**

The findings of this study will provide a basis for clinical judgment of whether there is a difference between the two forms of AF and PT reconstructed MPFL for RPD.

## 1. Introduction

Recurrent patellar dislocations (RPD) are often secondary or even multiple dislocations of the patella following re-trauma based on congenital dysplasia which belongs to the patellofemoral joint instability.^[1]^ Prevalent in obese adolescent females, this population frequently includes congenital pathological changes such as high patella, increased anterior femoral inclination, and femoral talus dysplasia.^[2–5]^ Epidemiological surveys have found that the incidence of patellar dislocation can be as high as 43/100,000 and is mostly associated with young women.^[6,7]^ The clinical symptoms are mainly pain (aggravated by going up and down stairs, squatting), instability (weakness, limpness), knee strangulation, recurrent swelling and movement disorders (afraid to run and jump or easily fall down).^[8]^ RPD is commonly treated with surgery, and different surgical approaches or a combination of surgical approaches can be chosen depending on the structural characteristics of the injury. However, the medial patellofemoral ligament (MPFL) can provide 50% to 80% of the inward restraining force to prevent lateral displacement of the patella and is the main stabilizing structure against lateral sliding of the patella, so RPD can be treated with the first choice of MPFL ligament reconstruction.^[3,9–11]^

The differences between the various MPFL reconstructions mainly lie in the fixation of the lateral patellar graft, which can be broadly classified into graft through patellar tunnel (PT) fixation and anchor fixation of the graft with sutures. Taking the graft through the patellar tunnel carries the risk of tunnel burst or even patellar fracture due to weakening of the bone cortex as a result of drilling,^[12–14]^ and because the graft needs to traverse the patellar tunnel, the required length of the graft increases accordingly, and more grafts may be required to accommodate this procedure,^[15]^ and the procedure may fail due to cuts to the tendon graft from the sharp tunnel opening. The sutured anchor fixation graft technique involves the placement of an endograft in a lateral patellar bone tunnel for fixation, but is relatively less invasive.^[16]^ With the same fixation strength,^[17]^ there are two distinct advantages: firstly, the anchor fixation technique reduces the potential risk of patellar fracture by creating a bone groove rather than a bone tunnel; secondly, it offers the possibility of reconstructing the MPFL for short tendons, since there is no need to fix the graft through a bone tunnel.^[18]^

Therefore, with the increasing number of comparative studies of these two fixation modalities, there is a lack of high-level guidelines and evidence-based medicine, which will have an impact on clinicians’ realistic decision making. Thus, we will conduct a meta-analysis to evaluate and compare the functional effectiveness of both fixation modalities, patellar anchor fixation graft (AF) and PT, in reconstructing the MPFL to treat RPD, and the conclusions drawn are expected to provide a reference to guide clinical practice.

## 2. Methods

### 2.1. Study registration

We have prospectively registered this research at the international prospective register of systematic reviews (PROSPERO)-Registration number: CRD42022373790. We performed this protocol based on the Preferred Reporting Items for Systematic Review and Meta-analysis Protocols (PRISMA-P) statement guidelines.^[19]^

### 2.2. Inclusion criteria

#### 2.2.1. Type of participants.

The participants diagnosed as RPD will be included regardless their country, ethnicity, sex, occupation and mechanism of injury.

#### 2.2.2. Type of interventions.

In the experimental group, all patients received MPFL reconstruction with patellar anchor fixation. In the control group, all patients received graft through patellar tunnel reconstruction of MPFL.

#### 2.2.3. Type of outcome measurements.

##### 2.2.3.1. Primary outcomes.

Re-dislocation rate.

##### 2.2.3.2. Secondary outcomes.

Validated knee function and activity scores such as Lysholm, return to former activities, Tegner score, visual analog scale (VAS) score, adverse events (complications), patient-reported instability symptoms, subsequent requirement for knee surgery (re-operations).

#### 2.2.4. Type of studies.

We will include comparative studies which published in Chinese or English, such as randomized controlled trials (RCTs), retrospective studies and cohort studies. Review, case reports, experimental studies, expert experience, animal studies and conference abstracts will be excluded.

### 2.3. Search strategy

CNKI, Wanfang, VIP, CBM, PubMed, Embase, and Cochrane Library databases were searched for this study. The search string is built as follows: (patellar dislocation OR dislocation OR medial patellofemoral ligament OR MPFL) AND (reconstruction) AND (anchor fixation OR anchor OR Bone tunnel OR tunnel). The search strategy in PubMed is shown in Table 1. In addition, the reference lists of previously published systematic reviews of Ligament Reconstruction for ankle instability were manually examined for further pertinent studies.

**Table 1:** Pubmed database search strategy.

Search number	Items
1	“ patellar dislocation “[Mesh]
2	patellar dislocation [Title/Abstract]
3	dislocation [Title/Abstract]
4	medial patellofemoral ligament [Title/Abstract]
5	MPFL[Title/Abstract]
6	1 OR 2 OR 3 OR 4 OR 5
7	reconstruction [Title/Abstract]
8	anchor fixation [Title/Abstract]
9	anchor [Title/Abstract]
10	Bone tunnel [Title/Abstract]
11	tunnel [Title/Abstract]
12	8 OR 9 OR 10 OR 11
13	6 AND 7 AND 12

### 2.4. Study selection

**Figure 1.**
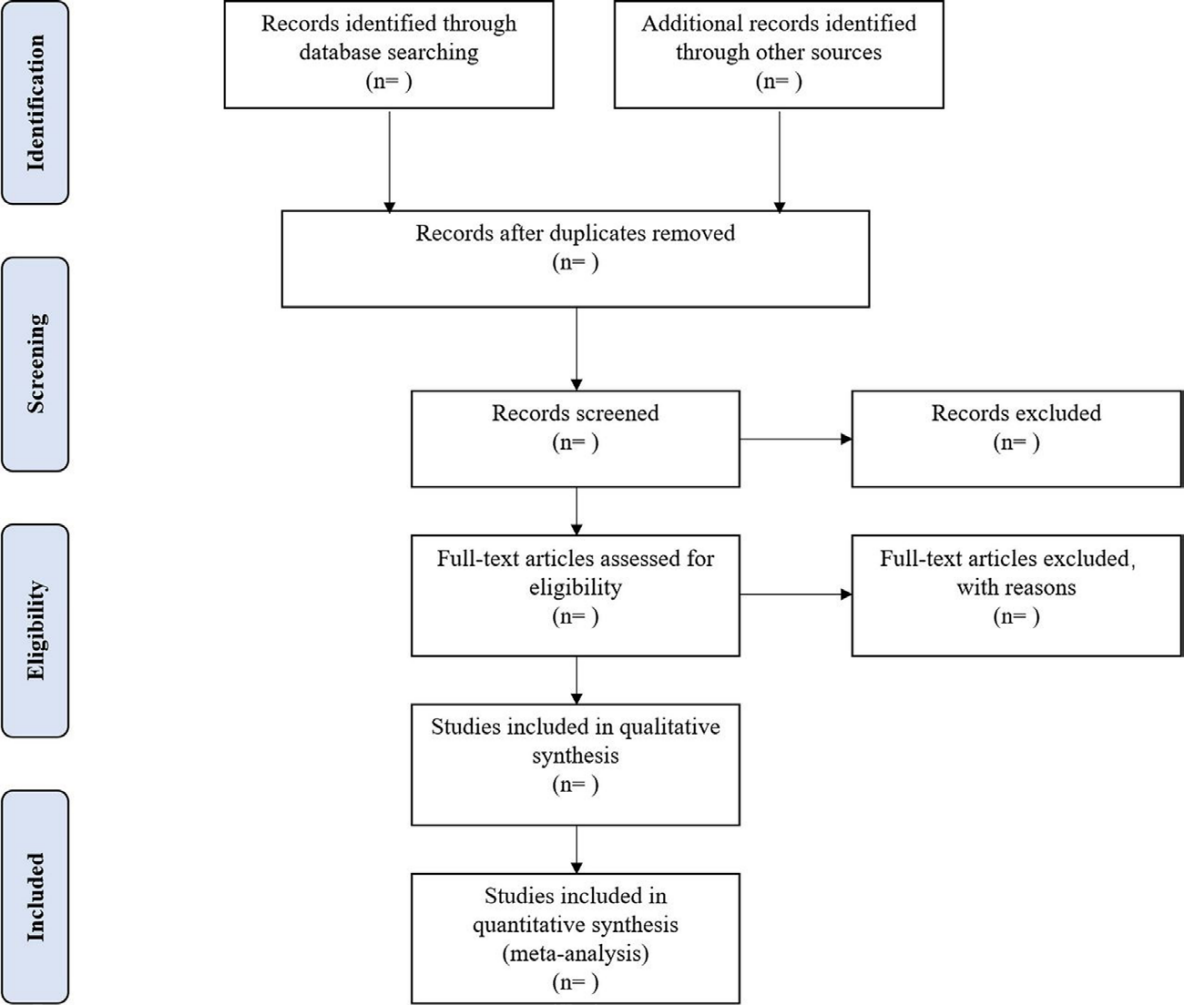
Flowchart of literature selection.

Different researchers will separately screen the titles and abstracts of records acquired potential eligibility which comes from the electronic databases. The obtained literature is managed by Notoexpress, irrelevant and duplicate articles are excluded by reading the title and abstract, Full texts screening and data extraction will be conducted afterward independently, and finally selected according to the inclusion criteria, Any disagreement will be resolved by discussion with the third author until consensus is reached or by consulting a third author. PRISMA-P flowchart will be used to show the selection procedure (Fig. 1).

### 2.5. Data extraction and management

The following data were extracted: lead author, publication year, country of origin, study design, sample size, age, injury type, fixation technique, re-dislocation rate, outcome measures and complications. Any differences of opinion will be resolved through group discussion or consultation with a third reviewer. When relevant data is not reported, we will contact the author via email or other means to obtain missing data. The Preferred Report items for the System Review and Meta-analysis (PRISMA) flow diagram will be filled out after the screening study is completed to provide specific information.

### 2.6. Risk of bias assessment

Two independent investigators evaluated the quality of the included studies. The Cochrane Collaboration Risk of Bias Tool was used to evaluate the quality of the RCTs. The methodological quality of the non-randomized studies was assessed using the Risk-of-Bias Assessment Tool for Non-randomized Studies (RoBANS). The level of evidence was assessed according to the Oxford Centre for Evidence-based Medicine Levels of Evidence.

### 2.7. Data synthesis

Statistical analysis will be conducted using RevMan 5.4 software (Cochrane Collaboration). The mean difference (MD) will be used as the effect analysis statistic for continuous variables, while the risk ratio (RR) will be used as the effect analysis statistic for categorical variables. We will also calculate 95% confidence interval (CI) for each statistic, and summarize statistical heterogeneity among summary data using the *I*^2^ statistic. Cases with *I*^2^ ≤ 50% will not be considered to have significant heterogeneity, thus a fixed-effects model will be applied for meta-analysis. In cases where there is statistical heterogeneity among studies, we will further analyze the source of heterogeneity. A random-effects model will be used to pool the data, after excluding the obvious source of clinical heterogeneity, and in cases where obvious clinical heterogeneity exists, the researchers will perform subgroup, sensitivity or only descriptive analyses. Study-specific and pooled estimates will be graphically presented using forest plots, and *P* < .05 considered statistically significant.

### 2.8. Subgroup analysis

If possible, we will analyze differences in primary and secondary outcomes in the following subgroups of patients: type of surgery, time from first dislocation to surgical treatment, number of dislocating events before surgery, physical activity level, presence of open or closed physic, age and sex.

### 2.9. Sensitivity analysis

Sources of heterogeneity were assessed by sensitivity analysis, by excluding studies of low quality or small sample size, if the heterogeneity did not change significantly, the results were robust. Otherwise, the excluded studies may have been source of heterogeneity.

### 2.10. Publication bias

In this study, fewer than 10 included studies were evaluated for publication bias using funnel plot, otherwise Egger regression test would be used.^[20,21]^

### 2.11. Ethics and dissemination

No ethical approval is required because the study will be a review of literature and will not obtain data from a single patient. We will publish our findings through a peer-reviewed journal.

## 3. Discussion

The aim of this research was to comparatively evaluate the functional effects and complications of reconstructing MPFL with two fixation modalities, AF versus PT, in the treatment of RPD. We integrated the most recent and comprehensive clinical evidence in this field, expecting to provide patients and clinicians with useful, high-grade evidence-based medical evidence to assist clinical decision-making.

## Author contributions

**Conceptualization:** Jian Tian, Wei Dong.

**Data curation:** Changfei Yuan, Jian Tian.

**Formal analysis:** Liu Weitong.

**Funding acquisition:** Jinlei li, Wei Dong.

**Investigation:** Xiaoyan Zhang.

**Methodology:** Changfei Yuan, Jinlei li.

**Project administration:** Jingfan Yang.

**Resources:** Xing Zhou.

**Supervision:** Jiankun Chen.

**Software:** Hong Yin.

**Validation:** Jingfan Yang, Wei Dong.

**Writing – original draft:** Jinlei li.

**Writing – review & editing:** Jian Tian.
